# Hepatitis B Splice-Generated Protein Antibodies in Syrian Chronic Hepatitis B Patients: Incidence and Significance

**DOI:** 10.5812/hepatmon.13166

**Published:** 2014-04-16

**Authors:** Nour Al-Hanafi, Fawza Monem

**Affiliations:** 1 Department of Biochemistry and Microbiology, Faculty of Pharmacy, Damascus University, Damascus, Syria; 2 Clinical Laboratories Department, AL-Assad Hospital, Damascus University, Damascus, Syria

**Keywords:** Hepatitis B, Chronic, Syria, HBSP protein, Hepatitis B virus

## Abstract

**Background:**

Previous studies have suggested hepatitis B splice-generated protein (HBSP), when expressed, is involved in the pathogenesis of HBV infection.

**Objectives:**

We aimed to evaluate anti-HBSP incidence and association with several HBV infection parameters in a group of Syrian chronic hepatitis B patients.

**Patients and Methods:**

Eighty treatment-naïve HBsAg-positive adult chronic hepatitis B patients' sera were included in our prospective targeted study. Liver function, virological and histological tests results were obtained from patients’ medical files. Three variants of a 20-mer HBSP-derived peptide were designed based on HBV genome sequences obtained from Syrian patients' sera (GenBank Accession No. JN257148-JN257217). Microtiter plate wells were coated with the synthetic peptides and used to detect anti-HBSP antibodies by an optimized indirect enzyme-linked immunosorbent assay (ELISA). Samples were considered positive when showed optical density (OD) values higher than the cut-off value for at least one peptide variant.

**Results:**

Seven out of eighty (9%) CHB patients were positive for anti-HBSP antibodies. Mean OD values were not significantly different between HBeAg-positive and -negative patients (P > 0.05). OD values showed weak positive correlation with ALT and AST values (P < 0.05), and weak to moderate positive correlation with liver biopsy staging ranks (P < 0.05). No significant correlation was revealed with viral load values or liver biopsy grading ranks (P > 0.05).

**Conclusions:**

We introduced an anti-HBSP antibodies ELISA, designed for locally circulating HBV strains. Correlation observed of Anti-HBSP with liver fibrosis staging regardless of viral replication and liver inflammation suggests anti-HBSP antibodies as possible indicator for HBV-associated liver fibrosis.

## 1. Background

HBV infection is a serious global health issue. More than 240 million chronic hepatitis B (CHB) patients worldwide are at high risk of death due to cirrhosis and hepatocellular carcinoma (HCC) (1). In Syria, HBV infection is intermediately endemic (5-7%) and genotype D is predominant (2). Hepatitis B virus (HBV) is a DNA retro-transcribing virus including a circular 2.3 kb-length partially double-stranded DNA (dsDNA) genome with four overlapping open reading frames (ORFs) (3, 4). Splicing events in the viral mRNAs that might be subsequently encapsidated and retro-transcribed giving rise to defective viral particles have been reported in chronic hepatitis B (CHB) infection (5-9). Consequently, splice-generated viral proteins might be produced. A viral 111 aa-length protein generated by a fusion of HBV polymerase N-terminal to a new open reading frame, and encoded by a singly spliced mRNA has been reported (10, 11). This immunogenic hepatitis B splice-generated protein (HBSP) has been detected in the liver biopsies of patients with active chronic hepatitis (10, 12) and its involvement in the liver disease pathogenesis has been suggested (13). Antibodies to HBSP have been found in CHB patients sera and anti-HBSP detection has been proposed as a marker of HBV-related disease (12).

## 2. Objectives

The present study aimed at designing a semi-quantitative enzyme-linked immunosorbant assay (ELISA) to detect antibodies to hepatitis B spliced protein, and evaluate anti-HBSP incidence and association with HBV infection parameters in a group of Syrian chronic hepatitis B patients.

## 3. Patients and Methods

### 3.1. Specimens

Our prospective targeted study recruited eighty treatment-naive HBsAg-positive adult patients diagnosed with chronic HBV infection by credentialed gastroenterologists. None of the CHB patients manifested co-infection with HCV, HDV or HIV (anti-HCV-negative, anti-HDV-negative and anti-HIV-negative), or were alcohol-consuming or immuno-suppressed. Liver function tests (ALT and AST), virological markers (HBeAg and HBV DNA) and histological analysis, which was assessed according to Scheuer's classification for grading and staging of chronic hepatitis (14), were performed within maximally 4-week period around our study serum sampling. All aforementioned tests results were obtained from patients’ medical files. Forty-six HBsAg-negative, anti-HCV-negative healthy adults were also enrolled to obtain control sera. After the ethical committee's approval, written informed consents were obtained and peripheral blood specimens were drawn from all patients and healthy individuals. All sera were kept in -80°C.

### 3.2. HBSP-Derived Peptide Synthesis

Seventy complete HBV genome sequences obtained from Syrian patients' sera (GenBank Accession No. JN257148-JN257217) were multiply aligned to the NCBI reference sequences of HBV genotype D using Clustal W2 (15, 16). Consequently, donor and acceptor splice sites were identified for each at nucleotide positions 2447 and 489, respectively. Amino acid sequences of hepatitis B splice-generated protein (HBSP) were accordingly inferred by conceptual translation, and the consensus HBSP sequence was obtained. SYFPEITHI epitope prediction (http://www.syfpeithi.de/) (17) and HLA Peptide Binding Predictions (http://www-bimas.cit.nih.gov/molbio/hla_bind/) (18) algorithms were used to analyze the binding affinity scores of overlapping 9-mer peptides derived from our consensus HBSP to the predominant HLAA*0201 and HLA-B*0702 molecules (19). Three variants of a 20-mer peptide (variant 1, LLLKEPLCIPPVAVPNLRTE; variant 2, LLLKEPLCIPPAAVPNLRTE and variant 3, LLLKEPLCIPPVAVQNLRTE) spanning aa 62 to 81 of the 111 aa-length HBSP and covering 80%-variability of the analyzed sequences were consequently selected, checked for sequence specificity using BLASTP (http://blast.ncbi.nlm.nih.gov/) then synthesized at >80% purity (Pi Proteomics, LLC, USA). 

### 3.3. Antigen Coating

High-binding polystyrene microtiter plate wells (R&D Systems, Minneapolis, USA‬) were coated with 1 µg of a synthetic peptide using carbonate buffer (pH = 9.6) at 4°C overnight. Separate wells were dedicated for each synthetic peptide.

### 3.4. Anti-HBSP Antibodies Detection

Indirect enzyme-linked immunosorbent assay (ELISA) was performed for all patients and control sera in duplicate for the three peptide variants. Fifty microliters of serum diluted 1:100 in PBS, 0.05% Tween, and 1% PVP (pH 7.4) were added to each well and incubated for 1 hour at 37°C. After washing with PBS and 0.05% Tween, 50 µL of anti-human IgG HRP conjugate (1:2500) (Promega, Minneapolis, USA) were added, incubated for 30 minutes at 37°C and washed. Fifty microliters of freshly prepared OPD substrate solution (0.4 mg/mL o-phenylenediamin (OPD), 0.5 μl/ml 30% H_2_O_2_ in 0.1 M citric acid adjusted to pH 5.0 by NaOH) were added and incubated for 15 minutes. Optical density (OD) was measured by a spectrophotometer at 490 nm. The cut-off value was determined for each peptide variant as the mean OD of all control sera plus 3-fold the standard deviation (SD). The grey zone ranged between the mean OD of all control sera plus 2-fold the standard deviation and the cut-off value. Samples were considered positive when showing OD values higher than the cut-off value for at least one peptide variant. 

### 3.5. Statistical Analysis

Mean OD values were compared using Student's t test and difference was considered significant when P < 0.05. Correlation of OD values with other serological, virological and histological markers was assessed using Pearson's and Spearman's correlation coefficients. All analyses were performed using Microsoft Excel 2010 and IBM SPSS Statistics 17.0 software (International Business Machines Corp., New York, USA).

## 4. Results

As shown in Table 1, mean ALT and AST activity levels in chronic HBV patients were 70 IU/L (range 10-546 IU/L) and 57 IU/L (range 10-373 IU/L), respectively. HBeAg was positive in 15 of 80 patients. HBV DNA was detected in 77 of 80 patients including 54 patients demonstrating active replication (viral load > 10^5^ copies/mL). Liver biopsy assessment was available for 40 of 80 patients showing no fibrosis (S0; 15 patients, 37.5%), enlarged, fibrotic portal tracts (S1; 7 patients, 17.5%), periportal/portal-portal septa with intact architecture (S2; 12 patients, 30%), or probable/definite cirrhosis (S4; 6 patients, 15%); no portal/periportal or lobular activity (G0; 3 patients, 7.5%), inflammation without necrosis (G1; 22 patients, 55%), mild necrosis (G2; 13 patients, 32.5%), or moderate necrosis (G3; 2 patients, 5%); and hepatic steatosis (3 patients, 7.5%).

**Table 1.** Liver Function, Virological, Histological and anti-HBSP Antibodies Tests Results of Enrolled Syrian CHB Patients ^a^

Test	Test Results	P Value ^b^
**Liver Function Tests (n = 80)**		
ALT	mean 70 IU/L (range 10-546 IU/L)	< 0.05 ^c^
AST	mean 57 IU/L (range 10-373 IU/L)	< 0.05 ^c^
**Virological Markers (n = 80)**		
HBeAg	positive: 15 (19)	> 0.05 ^d^
HBV DNA	positive: 77 (96)	
Viral load	active replication (> 10^5^ copies/mL): 54 (68)	> 0.05 ^e^
**Histological Analysis (n = 40) ** ^**f**^		
Fibrosis staging	S0: 15 (37.5%); S1: 7, (17.5%); S2: 12 (30%); S3: 0 (0%); S4: 6 (15%)	< 0.05 ^g^
Necroinflammation grading	G0: 3 (7.5); G1: 22 (55); G2: 13 (32.5); G3: 2 (5); G4: 0	> 0.05 ^e^
**Anti-HBSP antibodies ELISA (n = 80)**	positive: 7 (9) ^h^	
	grey zone 15 (19) ^i^	

^a^ Data are presented in No. (%).

^b^ Statistical analysis of anti-HBSP antibodies ELISA results in terms of optical density (OD) values.

^c^ OD values showed weak positive correlation with ALT and AST values (Pearson's r values ranged between 0.21 and 0.25; ALT, P = 0.023; 0.031 and 0.026 for variants 1, 2 and 3 respectively; AST, P = 0.012; 0.048 and 0.028 for variants 1, 2 and 3 respectively).

^d^ Mean OD values were not significantly different between HBeAg-positive and -negative patients (Student's t test, P = 0.14; 0.25 and 0.32 for variants 1, 2 and 3 respectively).

^e^ OD values showed no correlation with viral load values (Pearson's correlation coefficient; P = 0.31; 0.39 and 0.42 for variants 1, 2 and 3 respectively) or liver biopsy grading ranks (Spearman's correlation coefficient; P = 0.77; 0.92 and 0.84 for variants 1, 2 and 3 respectively).

^f^ According to Scheuer's classification for grading and staging of chronic hepatitis for only 40 CHB patients with available liver biopsy (20).

^g^ OD values showed weak to moderate positive correlation with liver biopsy staging ranks (Spearman's ρ values ranged between 0.29 and 0.45; P = 0.04; 0.006 and 0.002 for variants 1, 2 and 3 respectively).

^h^ The cut-off value was determined for each peptide variant as the mean OD of all control sera plus 3-fold the standard deviation (SD). Samples were considered positive when showing OD values higher than the cut-off value for at least one peptide variant.

^i^ The grey zone ranged between the mean OD of all control sera plus 2-fold the standard deviation and the cut-off value.

Cut-off OD values [mean + 3 SD] determined for peptide variants 1, 2 and 3 were 1.10, 1.18 and 1.16, respectively. Seven of eighty chronic HBV patients were positive for anti-HBSP antibodies, five of which were positive for all three peptide variants while the remaining two patients were positive for either variant one or variant three. Accordingly, anti-HBSP antibodies incidence rate was 9%. The lower limits of the grey zone [mean + 2 SD] determined for peptide variants 1, 2 and 3 were 0.93, 0.97 and 0.97, respectively. Fifteen of eighty (15/80) patients were in the grey zone including one (1/15) for all peptide variants, six (6/15) for variants two and three, three (3/15) for variant one, one (1/15) for variant two and four (4/15) for variant three. Mean OD values were significantly different between patients and control sera (P < .001) but not between HBeAg-positive and -negative patients (P > 0.05). OD values showed weak positive correlation with ALT and AST values (Pearson's correlation coefficients ranged between 0.21 and 0.25; P < 0.05), and indicated weak to moderate positive correlation with liver biopsy staging ranks (Spearman's correlation coefficients ranged between 0.29 and 0.45; P < 0.05). No significant correlation was revealed with viral load values or liver biopsy grading ranks (P > 0.05; Table 1).

## 5. Discussion

Our anti-HBSP antibodies semi-quantitative ELISA showed significant discrimination between chronic hepatitis B patients and the control sera (P < 0.001). However, a relatively low incidence rate (9%) of anti-HBSP antibodies among Syrian CHB patients enrolled in our study compared to previous studies (12) might be attributed to different technical conditions adopted including synthetic peptide variants and ELISA procedure. Furthermore, inability to detect the antibodies due to low antibody levels, antigen-antibody complex formation (21), or HBSP production down-regulation (20) might be speculated. Thus, anti-HBSP antibodies incidence rate might have increased if follow-up and retesting were accomplished for CHB patients showing negative or grey-zone results.

In the present study, no association was seen between anti-HBSP antibody detection and viral replication manifested by the viral loads and HBeAg status. HBSP hypothesized mechanism of action does not seem to impact viral replication (12). HBeAg production might be disabled by precore and basal core promoter mutations. Hence, predominant HBeAg-negative status in Syria does not necessarily indicate better prognosis or less replication (2). Conversely, Anti-HBSP antibodies status correlated moderately with fibrosis severity indicated by the liver biopsy staging ranks. This finding might be due to HBSP-induced apoptosis in the liver hepatocellular cells (22, 23), which might diminish the immune neutralization due to the viral particles spread (24). Accordingly, lack of/weak correlation of anti-HBSP antibodies status with necroinflammation grades/aminotransferase levels, respectively, is rationally explained. Furthermore, hepatic steatosis was found in three CHB patients' liver biopsies with fibrosis staging S1 (one patient) or S2 (two patients). However, this was overlooked since the association of liver steatosis with fibrosis severity in CHB infection is controversial (25-27). Our ELISA results using the three synthetic peptide variants were partially consistent. In particular, the peptide variant two could not detect all anti-HBSP positive sera. Moreover, the results of the peptide variant three showed the highest correlation coefficient with fibrosis severity. This might suggest variant three (LLLKEPLCIPPVAVQNLRTE) to be efficient for detecting anti-HBSP antibodies.

Albeit semi-quantitative, our assay paved the way to a tentative fibrosis assessment. Hence, developing the anti-HBSP ELISA described herein into a quantitative assay and evaluating its sensitivity and specificity empirically might be advisable to determine anti-HBSP antibodies levels discriminating different liver biopsy staging ranks. In conclusion, we introduced an anti-HBSP antibody ELISA, purposely designed based on genomic sequences obtained from HBV strains circulating in the Syrian population. Although anti-HBSP test did not show any significant association with parameters of viral replication and liver inflammation, it delivered partial indication on liver fibrosis associated with chronic hepatitis B.
